# Eph/Ephrins-Mediated Thymocyte–Thymic Epithelial Cell Interactions Control Numerous Processes of Thymus Biology

**DOI:** 10.3389/fimmu.2015.00333

**Published:** 2015-06-26

**Authors:** Javier García-Ceca, David Alfaro, Sara Montero-Herradón, Esther Tobajas, Juan José Muñoz, Agustín G. Zapata

**Affiliations:** ^1^Department of Cell Biology, Faculty of Biology, Complutense University, Madrid, Spain; ^2^Cytometry and Fluorescence Microscopy Center, Complutense University, Madrid, Spain

**Keywords:** thymocytes, thymic epithelium, Eph, ephrin, thymic cell seeding

## Abstract

Numerous studies emphasize the relevance of thymocyte–thymic epithelial cell (TECs) interactions for the functional maturation of intrathymic T lymphocytes. The tyrosine kinase receptors, Ephs (erythropoietin-producing hepatocyte kinases) and their ligands, ephrins (Eph receptor interaction proteins), are molecules known to be involved in the regulation of numerous biological systems in which cell-to-cell interactions are particularly relevant. In the last years, we and other authors have demonstrated the importance of these molecules in the thymic functions and the T-cell development. In the present report, we review data on the effects of Ephs and ephrins in the functional maturation of both thymic epithelial microenvironment and thymocyte maturation as well as on their role in the lymphoid progenitor recruitment into the thymus.

## Introduction

The thymus is a lymphoid organ engaged in the production and homeostatic maintenance of functionally mature T cells, in which developing thymocytes interact sequentially with an epithelial network whose three-dimensional architecture is essential for the process. Thymocyte–thymic epithelial cell (TEC) interactions are, therefore, key for thymus functioning (1), and Eph and ephrins, two groups of molecules involved in these cell-to-cell contacts, have emerged as novel elements governing numerous thymic processes (2). Eph represent the largest group of receptor tyrosine kinases; they bind to surface ligands, ephrins and, according to their sequence homology and affinity for ephrins, are divided into EphA (10 members), which preferentially binds ephrins-A (6 members), ligands bound to the membrane through glycosylphosphatidylinositol, and EphB (6 members) that bind ephrins-B (3 members) that contains a transmembrane domain and a short cytoplasmic tail (3).

Eph/ephrins constitute an ubiquitous system due to the high number of members and their promiscuity, such that a single receptor can bind different ligands and vice versa, albeit with distinct affinities (4). Eph/ephrin-mediated interactions result in bidirectional signaling in the expressing cells, *forward* signals transmitted by Eph, and *reverse* in the ephrin-expressing cell (5), providing different cell responses depending on the multiple combinations and the direction of signaling (4). Eph/ephrins activate numerous signaling pathways that regulate cytoskeleton and cell adhesion but also gene transcription (6).

## Eph and/or Ephrin are Expressed in the Thymus and Their Absence Results in Profound Thymic Hypocellularity

Eph and ephrins, particularly those of the B group, are expressed widely in both thymocytes and TECs, frequently the same cell co-expressing the two types of molecules. They appear early in the thymic primordium (7–9) and a lack of these results in decreased numbers of both thymocytes (9–11) and TECs (12). The thymic hypocellularity of Eph/ephrin-deficient mice correlates with concomitant increased apoptosis affecting distinct thymocyte subsets (9–11). However, in all these Eph/ephrin-defective mice, it is difficult to establish conclusive correlations between thymic cellularity and thymocyte proliferation. Even in thymocyte-conditioned ephrin-B1/B2 thymuses, despite the evident reduced cellularity, there are increased proportions of proliferating DP thymocytes (11), suggesting some attempt to recover the thymic cell content (13).

Thymic epithelial cells (TECs) have been proposed to have a limited expanding capacity and the number of endodermal progenitors that organize the early thymic primordium could determine the final size of embryonic and adult thymus (14). Indeed, there is little information on the control of TEC survival and proliferation in general, and by the EphB group in particular. Developing thymuses of EphB2- and/or EphB3-deficient mice show increased TEC apoptosis largely affecting immature EpCAM^+^MTS20^+^ cells and EpCAM^+^Ly51^+^ cTECs, and *in vitro* activation of either EphB or ephrin-B signaling decreases the proportions of apoptotic WT TECs, whereas its disruption in RTOCs resulted in increased TEC death. Importantly, RTOCs established only with EphB-defective TECs yielded higher proportions of apoptotic cells than those observed when RTOCs were established with TECs and total thymocytes, suggesting that TEC survival is governed to a greater extent by Eph–ephrin-mediated thymocyte–TEC interactions (12).

On the other hand, decreased seeding of lymphoid progenitors, which periodically colonize the thymus, could also contribute to organ size and cellularity. Reduced lymphoid seeding into the thymus can be achieved by a reduction in the colonizing progenitor numbers and/or altered mechanisms of migration. Although, EphB2-deficient mice show decreased proportions of early BM hematopoietic progenitors compared to WT mice (15) their contribution to thymic seeding is a matter of discussion (16). On the contrary, BM cells expressing molecules known to be involved in thymus seeding (i.e., CCR7, CCR9, CXCR4, PSGL1) (17, 18) neither exhibit significant changes in EphB-deficient mice (15) nor in the numbers of fetal liver CD45^+^PIRA/B^+^ precursors (unpublished data). Therefore, the lack of EphB affects the migratory capacity of progenitor cells rather than the proportions of colonizing cells.

*In vivo* and *in vitro* assays have demonstrated that the lack of EphB in either lymphoid progenitors or thymic stroma reduces thymic seeding in both fetal (19, 20) and adult mice (15). *In vitro* colonization of WT FTOCs by EphB2^−/−^ or EphB3^−/−^, but not EphB2-LacZ, Lin^−^ BM cells was significantly reduced compared to that shown by WT cells (19) and adult WT mice showed lower seeding into the thymus after *in vivo* injection of EphB-deficient Lin^−^ BM cells than WT ones (15). This inability of progenitors to enter both fetal and adult thymus seems to be dependent, at least partially, on a direct role of Ephs in regulating cell migration, as previously reported for the cell migration of peripheral lymphocytes (21–23). *In vitro* migration of mutant progenitor BM cells was significantly reduced through fibronectin, laminin, or chemokine gradients, with a more severe reduction in EphB2-deficient cells than in EphB2-LacZ counterparts. Moreover, EphB2 stimulation by coated ephrin-B1Fc proteins inhibited laminin- and fibronectin-governing migration as well as CXCL12, CCL21, and CCL25-induced chemotaxis, but EphB2-LacZ cells did not exhibit reduced migration (19). This indicates that the extracellular domain of EphB promotes migration ligand independently while forward signaling promotes cell arrest.

In both experimental approaches, all tested BM progenitors, included WT ones, showed decreased migration into the EphB-deficient thymus, particularly the EphB2^−/−^ one, which indicates the relevance of the thymic microenvironment in the process (15, 19). In fact, in both adult and fetal thymuses, decreased migration correlated with reduced production of ECM components, such as fibronectin and laminin, and chemokines (i.e., CXCL12, CCL21, CCL25) (15, 19). Furthermore, P-selectin involved in progenitor cell migration into the adult thymus (17), showed reduced expression on endothelial cells of both EphB2- and EphB3-deficient thymuses, but not of those of EphB2-LacZ cells, and decreased migration in EphB2^−/−^ thymuses also correlated with reduced endothelial expression of ephrin-B1 and ephrin-B2, whereas in EphB3^−/−^ thymuses, the reduction only affected ephrin-B1, reinforcing the idea that forward signals mediated by the pair EphB2/ephrin-B1 are particularly important for intrathymic lymphoid recruitment (15).

All these results, therefore, support a role for reduced thymic seeding in the thymic hypocellularity found in Eph/ephrin mutants. However, increased apoptosis of both thymocytes and TECs seems to be more relevant because, whereas thymuses deficient in both CCR7 and CCR9 with profoundly altered lymphoid colonization later recover normal thymocyte numbers (24), EphB2- and EphB3-deficient thymuses do not show that compensatory property; on the contrary, they increase their hypocellularity by increasing the death of DN and DP cells (9).

## Thymic Alterations Observed in Eph/Ephrin-deficient Mice Reflect the Relevance of Thymocyte–TEC Interactions

Eph/ephrin deletion results in specific phenotypic alterations in both thymocytes and TECs. The lack of EphA4 results in a blockade of T cell maturation that results in a drop in DP cell proportions (10) and blockade of Eph/ephrin-A interactions in FTOCs treated with fusion proteins affects the maturation of immature CD4^−^CD8^+^ thymocytes (2). In correlation, these thymuses show a profound collapse of the cortical epithelial network that significantly reduces the number of cell layers and their organization whereas immature K5^+^K8^+^ TECs and areas devoid of epithelial cell marker expression increase. Apparently, the epithelial defects determine the lymphoid phenotype because mutant FTOCs grafted under the WT kidney capsule produce decreased proportions of DP thymocytes (10) while mutant thymocytes in a WT stroma do not reproduce these changes. On the contrary, in Eph/ephrin-B-deficient mice, alterations are very important in the epithelial component but less severe in the developing thymocytes (9, 11, 25). EphB2- and EphB3-deficient thymuses exhibit minimal changes in the T-cell subset proportions, with an increased percentage of total DN cells and reduction of DN3 (CD44^−^CD25^+^) cells (9). However, in this case, EphB acts cell-autonomously on T-cell differentiation as grafted EphB-deficient alymphoid fetal thymus lobes colonized by WT lymphoid progenitors exhibit normal T-cell differentiation (26), while chimeric thymuses generated with EphB2^−/−^ and EphB2/B3^−/−^ Lin^−^ BM cell progenitors injected into SCID mice showed a blockade of T-cell maturation at DN stage and chimeras established with EphB3^−/−^ progenitor cells showed a partial blockade at this same point that resulted in low numbers of DP cells (27). Therefore, both EphB2 and EphB3 autonomously control thymocyte development at DN to DP transition. Both molecules are also necessary for the maturation of DP cells to SP thymocytes as demonstrated in reaggregates (RTOCs) formed with EphB2- or EphB3-deficient DP thymocytes and WT TECs (28). Eph expression on thymocytes is also important for thymocyte survival as in all these chimeric SCID mice there were increased proportions of apoptotic thymocytes, principally DP and SP CD4^+^ cells. Both thymocyte differentiation and survival seem to be dependent on Eph/ephrin-mediated thymocyte–TECs interaction and regulated by both forward and reverse signals, as SCID mice receiving EphB2-LacZ cells showed DP cells but did not produce SP thymocytes and did not show increased apoptosis. Therefore, although Eph forward signaling on thymocytes is necessary for thymocyte development, reverse signaling on interacting cells, presumably thymic epithelium, partially rescues DN cell progression to the DP cell compartment, and is important for cell survival (27). Accordingly, conditional deletion of ephrin-B1 and/or ephrin-B2 in TECs also affects the T-cell development and the lack of ephrin-B2 is presumably the most important, although ephrin-B1 also contributes, as double mutants show a more severe affectation (11). In addition, specific deletion of these ephrins in thymocytes results in a partial blockade of T-cell maturation at the DN3 stage (11, 29, 30) and increased thymocyte apoptosis (11). The phenotype is similar in single and double mutants suggesting that both molecules have a cooperative rather than redundant role in thymocyte maturation (11). A similar phenotype, however, has not been found when EphB2- or EphB3-deficient thymocytes are developed in a WT stroma in a bone marrow transplantation experiment into SCID mice (26).

Eph and ephrin signaling also affect thymic epithelium development and organization as in both EphB- and ephrin-B-mutant mice there is a profound transformation of thymic epithelium that exhibits altered TEC phenotypes (i.e., immature K5^+^K8^+^MTS10^+^ medullary epithelial cells (mTECs), cortical K5^−^K8^−^MTS20^+^ cells and K5^+^K8^+^ cells) and altered 3D organization. This change provokes a 2D structure that results in increased epithelial cysts, collapsed epithelium, and large areas devoid of epithelial cell markers (11, 25, 31). These latter areas, of unknown significance, also exist in WT thymuses and in other mice with defects in molecules, such as Foxn1, Kremen 1, or Stat3, involved in TEC maturation (32–34), but are specially developed in EphB-deficient thymuses. They contain thymocytes and blood vessels, frequently surrounded by enlarged sheaths of connective tissue, and are different in cortex and medulla: the former ones contain thymocytes and some sheathed blood vessels, whereas in the medulla mTECs delimit areas with enlarged blood vessels, increased numbers of ER-TR7^+^ fibroblasts, components of the ECM (collagen IV, fibronectin, and laminin) (35), dendritic cells (36), and thymocytes in some areas (37–39).

Cortical areas devoid of epithelium have been described by others (37–39), receiving the name of epithelial-free areas (EFAs). EFAs are MHC class-II negative, little vascularized areas that contain abundant thymocytes frequently in division (39) reported as accumulations of DP thymocytes that do not undergo positive selection and will die subsequently by apoptosis (40). On the contrary, medullary epithelium-free areas that express several connective tissue markers could have a mesenchymal condition (35) and represent areas in which Eph-deficient TECs have undergone an epithelial–mesenchymal transition, losing their epithelial cell markers and acquiring a mesenchymal nature. In Eph mutant mice, EFAs could arise as a consequence of impeded intermingling and mutual exclusion of thymocytes and TECs caused by the lack of Eph–ephrin signaling as known in other systems (41).

Presumably, TEC maturation is autonomously governed by EphB2 and EphB3 expressed on TECs, as some of the phenotypical alterations found in EphB2- or EphB3-deficient mice can be reproduced in grafted mutant lobes colonized by WT host thymocytes. However, EphB expressed on thymocytes can also play a non-autonomous role since the epithelial phenotype of these grafted mutant lobes was not exactly the same as that found in EphB-deficient thymuses (26), and chimeric SCID thymuses receiving EphB-deficient thymocytes showed altered histological organization (27).

Selective deletion of ephrin-B1 and/or ephrin-B2 genes in thymocytes or TECs permits to determine the relevance of Eph/ephrin signaling in distinct thymic components (11). In all ephrin-B-deficient mice, but particularly in the double mutants with ephrin-B1 and ephrin-B2 deleted in TECs, the thymuses are small, with scarcely developed cortex and medulla, high numbers of K5^+^K8^+^ cells, and numerous epithelial cysts. Ephrin-B2 deletion in TECs causes altered distribution of Ly51^+^ cortical (c) TEC subsets defined as Ly51^hi^ cells that express DLL4, and would constitute the cortical niche of DN thymocytes and Ly51^lo^ cTECs that would represent that of DP cells. Thus, ephrin-B1 deletion in TECs but, also, the lack of ephrin-B1 and B2 in thymocytes induce a cortex in which rounded groups of cTECs express Ly51 homogeneously, suggesting that ephrin-B1 and B2 expressed in both thymocytes and TECs cooperate in regulating the differentiation and distribution of cortical niches (11). Furthermore, ephrin-B deletion from both thymocytes and TECs affects medulla organization. The medulla of thymuses bearing a deletion of ephrin-B1 or B2 in thymocytes, or those without ephrin-B1 or ephrin-B1/B2 in TECs, shows increased numbers of large monolayered epithelial cysts formed largely by immature K5^+^K8^+^, sometimes MTS20^+^, TECs, but rarely containing mature UEA1^+^ or MTS10^+^ cells that would represent an arrest of medulla development at an immature stage. They also contain numerous UEA1^hi^ cells that form small cysts surrounded by a thin rim of UEA1^lo/−^MTS10^+^ cells that would represent a certain blockade of medulla organization at a late stage of development, in which medullary TEC subsets UEA1^hi^MTS10^−^ and UEA1^lo^MTS10^+^ develop but MTS20^+^ cell expansion and 3D organization are affected (31).

Interestingly, these studies support that ephrin-B1 and ephrin-B2 deletion in TECs result in different TEC phenotypes similar to those generated by ephrin-B1 or ephrin-B2 deletion in thymocytes. This, once again, indicates that Eph/ephrin-mediated thymocyte–TEC interactions are also important for TEC development and arrangement. Although, these molecules also mediate homotypic interactions (thymocyte–thymocyte; TEC–TEC), presumably their involvement in thymocyte–TEC interactions is more important to explain their role in the thymus. However, there are no complementary phenotypes when the effects of ephrin deletion in TECs are compared with those observed in thymuses with EphB-deficient epithelium, or when Eph mutant or ephrin mutant phenotypes are compared. This indicates that in the thymus, as in other systems (4), the final balance of *forward* and *reverse* signals in thymocytes and/or TECs would be more relevant than the mere presence/absence of certain Eph or ephrins. Besides, other factors must contribute to the complexity of the system as phenotypes in different mutant models are more severe in mice with C57/Bl6–CD1 mixed background than in the non-inbreed strain C57/Bl6 (10, 11).

*In vitro* experiments also clearly support the relevance of Eph/ephrin-mediated thymocyte–TEC interactions in thymus biology: ephrin-B1Fc proteins added to RTOCs, formed by fetal TECs and DP thymocytes, disorganize the 3D thymic epithelial network, prevent thymocyte–TEC association, and alter TCRαβ signaling (28). Numbers and timing of the establishment of cell conjugates also change when they are established with EphB-deficient DP thymocytes (27).

On the other hand, proper T-cell maturation occurs thanks to the movement of developing thymocytes throughout the thymic parenchyma, facilitating their interactions with distinct niches favoring the necessary thymocyte–TEC crosstalk (42). Analysis by confocal microscopy of the positioning of EphB2-deficient or WT Lin^−^ BM progenitors in reconstituted FTOCs demonstrated that higher numbers of WT cells reached the central area of WT lobes than of EphB2-deficient cells (19). Furthermore, EphB2^−/−^ total thymocytes migrate less efficiently through laminin or fibronectin or in response to CXCL12, CCL21, or CCL25, than WT cells. More importantly, when *forward* EphB2 signals were activated by ephrin-B1Fc protein treatment, there was a significant reduction in the migration of all EphB2^−/−^, but not EphB2-LacZ, thymocyte subsets. Therefore, together with chemokines and ECM molecules, the migration of developing thymocytes throughout the thymic stroma could be promoted by inactivated EphB2 receptors, and negatively modulated by EphB2/ephrin-B interactions (19).

Remarkably, these profound phenotypic alterations observed in mice deficient in distinct Eph or ephrins do not correlate with immune deficiencies and/or pathological processes. EphA4^−/−^ thymuses (10) and those with deleted ephrin-B1 and/or ephrin-B2 in TECs (11) show decreased proportions of both DP TCRαβ^hi^ cells and CD69^+^ cells that could reflect an inefficient TCRαβ selection. However, peripheral lymphoid organs of both EphA4^−/−^ mice (10) and EphB-deficient mice (9) show decreased numbers of total T cells, but not significant changes in the proportions of distinct T-cell subsets. In addition, there are no changes in the central and peripheral TCRαβ repertoire expressed on CD4^+^ T cells of EphB2- and/or EphB3-mutant mice, except for an increased proportion of Vβ3^+^CD4^+^ cells in both thymus and lymph nodes of the three mutants (43).

In summary, Eph and ephrins are molecules that through mediating thymocyte–TEC interactions are involved in numerous processes occurring into the thymus, including cell migration into and through thymus, T-cell differentiation, and TEC maturation.

## Conflict of Interest Statement

The authors declare that the research was conducted in the absence of any commercial or financial relationships that could be construed as a potential conflict of interest.
